# Supplementary material for: Can We Quantify Aging-Associated Postural Changes Using Photogrammetry? A Systematic Review
Source: Sensors (Basel). 2022 Sep 2;22(17):6640. doi: 10.3390/s22176640 (PMC9459795; doi:10.3390/s22176640)
Supplement: Supplementary file 1 [file sensors-22-06640-s001.zip › Document S1.pdf]

Search Strategy

Systematic Searches were conducted in March 2021 on PubMed, Embase, Scopus and SciELO.

The search terms and Boolean operators used in the searches are:

Photogrammetry AND Posture

Only studies in English were be included; in addition, manual searches were conducted on the references of the included studies.
