# Supplementary material for: Can We Quantify Aging-Associated Postural Changes Using Photogrammetry? A Systematic Review
Source: Sensors (Basel). 2022 Sep 2;22(17):6640. doi: 10.3390/s22176640 (PMC9459795; doi:10.3390/s22176640)
Supplement: Supplementary file 1 [file sensors-22-06640-s001.zip › Table S1.pdf]

| Study | Variable name |
|---|---|
| Drzal-Grabiec et al., 2012 | KNT (Coronal angle of trunk inclination) |
| | KPT (Sagittal angle of trunk inclination) |
| | UL (Height difference between lower scapular angles) |
| | UB (Depth difference between lower scapular angles) |
| | OL (Difference in distance between lower scapular angle and spine) |
| | KLB (Angle of shoulder line inclination) |
| | ALFA (Lumbosacral inclination) |
| | BETA (Thoracolumbar inclination) |
| | GAMMA (Upper thoracic inclination) |
| | KLL (Angle of lumbar lordosis) |
| | GLL (Depth of lumbar lordosis) |
| | KKP (Angle of thoracic Kyphosis) |
| | GKP (Depth of thoracic kyphosis) |
| | UK (Maximum deviation of spinous processes from the C7-S1 line) |
| Drzal-Grabiec et al., 2013 | KNT (Coronal angle of trunk inclination) |
| | KPT (Sagittal angle of trunk inclination) |
| | UL (Height difference between lower scapular angles) |
| | UB (Depth difference between lower scapular angles) |
| | OL (Difference in distance between lower scapular angle and spine) |
| | KLB (Angle of shoulder line inclination) |
| | ALFA (Lumbosacral inclination) |
| | BETA (Thoracolumbar inclination) |
| | GAMMA (Upper thoracic inclination) |
| | KLL (Angle of lumbar lordosis) |
| | GLL (Depth of lumbar lordosis) |
| | KKP (Angle of thoracic Kyphosis) |
| | GKP (Depth of thoracic kyphosis) |
| | UK (Maximum deviation of spinous processes from the C7-S1 line) |
| Drzał-Grabiec et al., 2014 | GKP (Depth of thoracic kyphosis) |
| | GLL (Depth of lumbar lordosis) |
| | KLB (Angle of shoulder line inclination) |
| | UB (Depth difference between lower scapular angles) |
| | UL (Height difference between lower scapular angles) |
| | UK (Maximum deviation of spinous processes from the C7-S1 line) |
| Wild et al., 2014 | Pelvic Obiquity |
| | Pelvic Torsion |
| | Surface Rotation |
| | Lateral Deviation |
| | Kyphosis Angle |
| | Lordosis Angle |
| Gong et al., 2019 | Neck |
| | Thorax |
| | Waist |
| | Hip |
| | Knee |
